# A Study on the Orientation Relationship and Interface Structure of the α_2_ (Ti_3_Al) and B2 Phases in the TiAl-Nb Sheets After Heat Treatment

**DOI:** 10.3390/ma19112427

**Published:** 2026-06-05

**Authors:** Jiyao Liu, Laiqi Zhang, Muyu Li, Dan Yao, Yixu Niu, Bin Li, Yahu Song

**Affiliations:** 1 School of Intelligent Manufacturing, Luoyang Institute of Science and Technology, Luoyang 471023, China; 2 Henan International Joint Laboratory of Cutting Tools and Precision Machining, Luoyang Institute of Science and Technology, Luoyang 471000, China; 3 State Key Laboratory for Advanced Metals and Materials, University of Science and Technology Beijing, Beijing 100083, China; 4 School of Intelligent Vehicle Engineering, Luoyang Institute of Science and Technology, Luoyang 471023, China

**Keywords:** TiAl, γ phase, α2 phase, heat treatment, mismatch, orientation relationship

## Abstract

In this paper, TiAl-Nb sheets were fabricated via elemental foil metallurgy using Ti, Al, and Nb foils. The microstructure of the TiAl-Nb sheet was regulated by a two-step heat treatment process, which involved short-time holding at 1410 °C, 1430 °C and 1450 °C, followed by long-time holding at 1150 °C. Subsequently, the microstructure of the sheet was analyzed, emphasizing the orientation relationship and interface structure between the B2/β phase, Ti_3_Al phase, and the TiAl matrix. The results indicated that, subsequent to diverse heat treatment processes, the TiAl-Nb sheet comprised α_2_(Ti_3_Al) and B2/β phases at the grain boundaries and within the grains, whereas the matrix structure was γ(TiAl). After TiAl-Nb sheets were heat-treated at 1410 °C for 3 min and then at 1150 °C for 2 h, the microstructure of the sheets was observed to be composed of relatively fine lamellar structures. The TiAl phase, Ti_3_Al phase and B2/β phase existed in the form of coherent interfaces with extremely small misfit degrees. The interfacial energy between phases was small, making it easier to obtain an alloy microstructure with a higher content of the γ(TiAl) phase. To further provide a basis for the selection of heat treatment processes, the matrix method analysis indicated that, after holding at 1410 °C for 3 min and subsequently at 1150 °C for 2 h, the TiAl phase and Ti_3_Al phase in the sheet structure exhibited obvious preferred orientations. A short β-phase holding (3 min) followed by a long α + γ two-phase holding was an effective process route for obtaining a fine lamellar structure.

## 1. Introduction

As a novel type of lightweight and high-temperature resistant material, TiAl alloy has been effectively utilized in low-pressure turbine blades and other structures of high-performance engines owing to its outstanding properties (such as low density, high oxidation resistance, creep resistance, and high-temperature strength) [1,2,3], thereby enhancing overall aircraft performance [4,5]. Owing to the intrinsically low room-temperature plasticity and inferior formability of TiAl alloys, their practical utilization in diverse significant fields has been seriously impeded [6]. Currently, within the aerospace industry, substituting nickel-based superalloys with cost-effective TiAl alloys at temperatures exceeding 750 °C still poses a challenge [7,8].

To address the intrinsic brittleness of TiAl intermetallic compounds, there are currently two primary approaches for enhancing their brittleness: one is alloying, and the other is heat treatment. By introducing β-stabilizing elements typified by Nb, the α-phase region is narrowed. When considered in conjunction with the phase diagram, the alloy microstructure shifts towards higher temperatures and higher Al content, thereby enhancing the service temperature, high-temperature creep resistance, and oxidation resistance of TiAl alloys [9,10]. Heat treatment exerts a substantial influence on the microstructure of materials. Through the formulation of a rational heat treatment process, the microstructure of TiAl alloys could be made more uniform. Additionally, it has a refining effect on the lamellar structure within the microstructure [11], thereby enabling the alloys to attain more outstanding room-temperature performance [12,13].

During the heat treatment process of TiAl alloy sheets, phase transformation and the formation of layered structures are crucial. The microstructure has a significant impact on the mechanical properties of TiAl alloys. Chlupova et al. [14] examined the impact of heat treatment on the microstructure and fatigue performance of layered high Nb-TiAl alloys. The findings demonstrated that, via high-temperature heat treatment, the microstructure of the material was substantially refined, and the layer spacing was considerably decreased. The reduction in layer spacing necessarily resulted in an increase in grain boundary density, thereby enhancing the strength at both room temperature and high temperatures. Yue et al. [15] conducted an investigation into the influence of heat treatment on the microstructure and tensile properties of TiAl alloys. The findings demonstrated that high-temperature heat treatment would hinder the growth of elongated grains, ultimately resulting in the formation of nearly equiaxed grains, and the tensile anisotropy of the material was reduced. Liu et al. [16] discovered that, subsequent to four cycles of cyclic heat treatment being applied to the Ti44Al alloy, the microhardness exhibited a 28% increase in comparison to the as-cast structure. Varying heat treatment temperatures and holding times can influence microstructure morphology, lamellar size, and mechanical properties post-heat treatment. However, these studies solely concentrated on the traditional TiAl alloys encompassing the γ, α_2_, and β/B2 phases. Significantly, there remains a paucity of information concerning the phase interface structure and orientation relationship in TiAl alloys with (γ + β) phases.

The preparation conditions for TiAl alloy sheets were highly rigorous, necessitating special rolling processes like isothermal rolling. This notably elevated the process cost and manufacturing complexity [17,18]. The elemental foil metallurgy method [19] primarily fabricated TiAl alloy sheets via the chemical reaction between Ti and Al elements [20], thereby circumventing the direct processing of TiAl alloys. Therefore, in this paper, TiAl-Nb sheets were initially fabricated using the elemental foil metallurgy, and subsequently, the microstructure of the sheets was adjusted through heat treatment based on the thermal analysis data. The nominal composition of the alloy investigated in this study was Ti-47Al-5Nb (at.%), corresponding to a Ti atomic fraction of 48 at.%. Accoring to the Ti-Al binary phase diagram, an Al content of 46–48 at.% promoted the formation of a γ-TiAl + α_2_-Ti_3_Al duplex microstructure at intermediate and high temperatures, and facilitated the development of a fine lamellar microstructure after appropriated heat treatment. A higher Ti content (e.g., >50 at.%) led to an excessive volume fraction of the α_2_ phase and reduced room-temperature ductility, whereas a lower Ti content (<45 at.%) destabilized the γ phase and deteriorated high-temperature oxidation resistance. Chlupova et al. [14] investigated a Nb-containing TiAl alloy (Ti-46Al-8Nb, Ti ≈ 46 at.%). After heat treatment, the lamellar spacing decreased from 150 nm to 50 nm, accompanied by a 30% increase in strength. These findings highlighted the importance of optimizing the Ti content within the range of (46–48 at.%) in combination with suitable heat treatment conditions.

The microstructure and phase of the heat-treated sample were analyzed via SEM and XRD. The interfaces of the α_2_(Ti_3_Al), B2, and γ(TiAl) phases were observed through EBSD, TEM, and HRTEM. The orientation relationship and structural stability of the phase interfaces in the heat-treated sheet were investigated using the mismatch theory and matrix method. Ultimately, a microstructure consisting of the γ phase and α_2_/γ lamellar structure was obtained. This study systematically compared the effects of short β-phase holding times (3 min and 5 min) on the lattice misfit and preferred orientation relationships of γ/α_2_/B2 interfaces, thereby establishing correlations between processing parameters and interfacial characteristics through cross-validation using EBSD pole figures and HRTEM diffraction patterns. The findings of this study offer valuable guidance for the control of microstructures and the enhancement of performance, and also act as a foundation for the selection of heat treatment processes.

## 2. Materials and Method

### 2.1. Sample Preparation

The raw materials used in this experiment were Ti foil (purity 99.9%, thickness 0.05 mm; Beijing Cuibolin Metal Materials Co., Ltd., Beijing, China), Al foil (purity 99.9%, thickness 0.05 mm; Beijing Cuibolin Metal Materials Co., Ltd., Beijing, China) and Nb foil (purity 99.9%, thickness 0.04 mm; Hebei Qingyuan Metal Materials Co., Ltd., Hebei, China). Ti foil, Al foil and Nb foil, with thicknesses of 0.05 mm, were cut into 40 mm × 50 mm square pieces. The surfaces of Ti foil, Al foil, and Nb foil were ground using 400 mesh sandpaper (Yingpai, Hubei Yuli Abrasive Belt Group Co., Ltd., Tongcheng, Hubei, China) to eliminate the oxide layer. Subsequently, they were immersed in an alcohol solution and subjected to ultrasonic cleaning for 10 min. Cleaned titanium (Ti) foil, aluminum (Al) foil, and niobium (Nb) foil were cross-stacked at a Ti-47Al-5Nb (at%) ratio to form a “sandwich” structure with a thickness of approximately 5 mm and subsequently cold-rolled. The rolling process of the Ti/Al/Nb composite sheet was carried out as follows: the deformation amount per single pass was 0.5 mm, the rolling speed was 0.16 m/s, and the total deformation amount reached 62.5%. The final thickness of the sheet was approximately 2 mm. Subsequently, they were placed in the designed stainless steel can for the diffusion reaction. The can was welded and sealed under an argon gas atmosphere. The diffusion reaction was carried out under the following conditions: 1000 °C for 48 h followed by 1400 °C for 24 h, and then air-cooled. After removing the can, the sheet surface was ground with sandpaper to remove any possible diffusion layer and trace oxide scale. Finally, the TiAl-Nb sheet was cut into circular pieces with a diameter of 50 mm. The specimens were placed in the SPS (spark plasma sintering furnace, model SPS4-20T-101V, Shanghai Chenhua Technology Co., Ltd., Shanghai, China), sintered at 1100 °C under a pressure of 50 MPa, and held under this pressure for 20 min for densification treatment.

### 2.2. Heat Treatment Process and Microstructure Analysis

**Figure 1:**
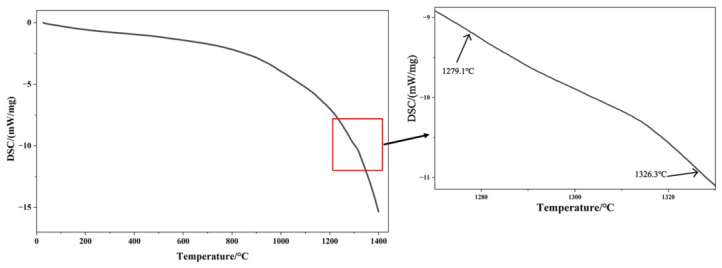
DSC curve of TiAl-Nb sheet.

Based on the DSC curve (Figure 1) and the Ti-Al-Nb phase diagram, a short holding treatment at 1410–1450 °C with the β single-phase region for 3 min was selected to suppress excessive α-phase growth. This treatment was followed by a prolonged holding at 1150 °C within the α + γ region for 2 h to enable homogeneous Nb diffusion and complete transformation into a fine γ + α_2_ lamellar structure. The target microstructure consisted of a γ-TiAl matrix containing a fine α_2_/γ lamellar structure and dispersed B2/β particles, characterized by coherent or semi-coherent interfaces with low lattice misfit between adjacent phases. The TiAl-Nb sheets that had undergone densification treatment were subjected to microstructure regulation via heat treatment in accordance with the processes listed in Table 1. The heat treatment structure was controlled using a tube furnace (model GSL-1700, Hefei Kejing Materials Technology Co., Ltd., Hefei, China) with an argon atmosphere. The heating rate was 10 °C/min. During the cooling process, it was first reduced from the heat treatment temperature at a rate of 5 °C/min to 500 °C, and then cooled in the furnace. Subsequently, the sheets were cut into specimens for SEM and XRD tests. SEM observation was performed using a field-emission scanning electron microscope (model SUPRA55, Carl Zeiss AG, Oberkochen, Germany). XRD analysis was conducted using an X-ray diffractometer (model TTR3, Rigaku Corporation, Tokyo, Japan). The transmission electron microscope sample was prepared using the double-spray thinning method. The double-spray etching solution consisted of perchloric acid (Sinopharm Chemical Reagent Co., Ltd., Shanghai, China), methanol (Sinopharm Chemical Reagent Co., Ltd., Shanghai, China), and n-butanol (Sinopharm Chemical Reagent Co., Ltd., Shanghai, China), at a volume ratio of 5:60:35. The double-spray temperature ranged from −20 °C to −30 °C, and the voltage was set at 25 V. The double-spray thinning instrument used was a TenuPol-5 (Struers A/S, Ballerup, Denmark).

The EBSD sample was initially ground using sandpaper, progressing until 2000# sandpaper (Yingpai, Hubei Yuli Abrasive Belt Group Co., Ltd., Tongcheng, Hubei, China) was employed. The electrolyte utilized for electrolytic polishing consisted of perchloric acid (Sinopharm Chemical Reagent Co., Ltd., Shanghai, China), n-butanol (Sinopharm Chemical Reagent Co., Ltd., Shanghai, China), and methanol (Sinopharm Chemical Reagent Co., Ltd., Shanghai, China), with a volume ratio of 1:7:12. Throughout the electrolytic polishing procedure, liquid nitrogen was introduced into the electrolyte to maintain the temperature within the range of −30 °C to −20 °C. Subsequently, a constant voltage of 30 V was applied for 25 s. EBSD analysis was performed using the same field-emission scanning electron microscope (model SUPRA55, Carl Zeiss AG, Oberkochen, Germany) equipped with an EBSD detector.

## 3. Results and Discussion

To acquire TiAl-Nb alloy sheets with well-defined properties, it was essential to perform heat treatment and microstructure regulation on the TiAl-Nb sheets fabricated via the elemental foil metallurgy method. This enabled the elements within the microstructure of the TiAl-Nb alloy sheets to undergo further diffusion, thereby attaining the desired microstructure. Generally, heat treatment conducted at a temperature exceeding T_α_ led to the formation of a fully lamellar structure. Combining the DSC curve (Figure 1), the microstructure of Ti-47Al-5Nb sheets was regulated by formulating different heat treatment processes. To reveal the solid-state transformation peaks clearly, the DSC temperature range was narrowed to 1240–1340 °C and the baseline was corrected. The revised DSC curve showed distinct endothermic peaks at 1279.1 °C and 1326.3 °C, corresponding to the α→β and γ + α→β transformations, respectively. A systematic study on the phase transformation and lamellar formation mechanism during the heat treatment microstructure regulation process was conducted, with the ultimate goal of obtaining a microstructure composed of γ phase and α_2_/γ lamellar clusters.

During the heat preservation and furnace cooling process, it was also the formation process of the lamellar structure. In this process, primarily the α phase was formed, followed by a phase transformation. As the heat-preservation temperature lay within the β phase region, the α phase nucleated and grew at the grain boundaries and within the grains of the γ phase during the heat-treatment process. Subsequently, an increasing number of α and γ phases were formed and grew in the alloy’s microstructure. During this process, the α phase also underwent an ordered transformation and was converted into the α_2_ phase. After the heat treatment, a lamellar structure was finally formed in the microstructure [21]. As the holding time within the β phase region was extended, a greater number of α phases emerged in the microstructure, and the α phase underwent an ordered transformation to form a relatively coarse α_2_ phase. Therefore, during the subsequent optimization process of the heat treatment process, a method involving short-time holding at a high temperature and long-time holding at a low temperature could be employed to prevent the growth of the α_2_ phase in the microstructure and refine the lamellar structure. Long-time holding at a low temperature enabled the uniform diffusion of the Nb element.

### 3.1. Microstructure Analysis of TiAl-Nb Alloy Sheet After Heat Treatment

**Figure 2:**
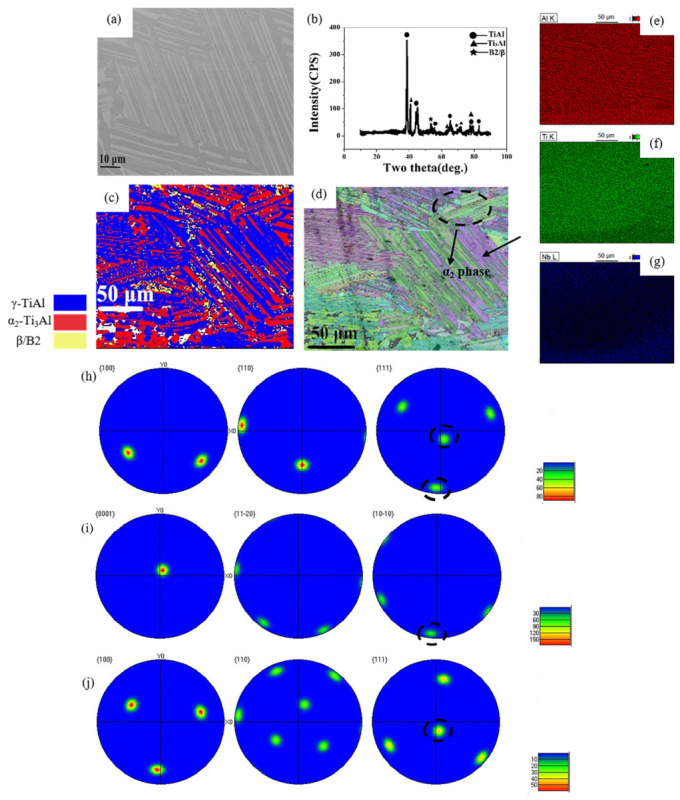
Microstructure, XRD pattern and EBSD characterization of the TiAl-Nb sheet after heat treatment at 1410 °C for 5 min and 1150 °C for 2 h ((**a**) microstructure; (**b**) XRD pattern; (**c**) phase distribution map; (**d**) microstructure under EBSD; (**e**–**g**) distribution of elements in the sheet; (**h**–**j**) pole figure of TiAl phase, Ti_3_Al phase and B2 phase).

Through the analysis of Figure 2a,b, it was found that, after heat treatment at 1410 °C for 5 min and then at 1150 °C for 2 h, the TiAl phase, Ti_3_Al phase and B2/β phase were the main phase components of the alloy. Although a short high-temperature holding (3–5 min) alone was insufficient for full Nb homogenization, the subsequent long holding at 1150 °C for 2 h effectively promoted Nb diffusion, as confirmed by EDS elemental mapping showing a uniform Nb distribution after the two-step treatment (Figure 2e–g). After heat treatment, lamellar structures were formed in the microstructure of the sheet. Since there were still some α_2_ phases that had not transformed into a finer lamellar structure within the microstructure, it suggested that the holding time at a higher temperature was relatively long. During the heat-treatment process, a significant amount of the α phase appeared in the structure. Subsequently, the already-grown α phase underwent an ordered transformation to form the α_2_ phase, leading to the formation of relatively coarse lamellar structures within the microstructure. Phase analysis and grain boundary analysis of the sheet were conducted by EBSD technology (Figure 2c,d). All EBSD phase distribution maps included a color code (blue for γ, red for α_2_, yellow for B2/β) for easy identification of each phase. An obvious lamellar structure was observed, but the lamellar structure was relatively coarse. The B2 phase was uniformly dispersed throughout the structure. The α_2_ phase predominantly existed within the γ phase, and there were also certain α_2_ phases that had not fully developed within the γ phase. Moreover, relatively coarse α_2_ phases were found at the grain boundaries of the γ phase. Quantitative EBSD analysis revealed that the volume fraction of the B2/β phase was approximately 3.5–5.2%, which was consistent with the relatively low diffraction peak intensity of this phase in the XRD pattern. The orientation relationships were as follows (Figure 2h–j): TiAl(111)//Ti_3_Al(10$\bar{1}$0), TiAl(111)//B2(111).

**Figure 3:**
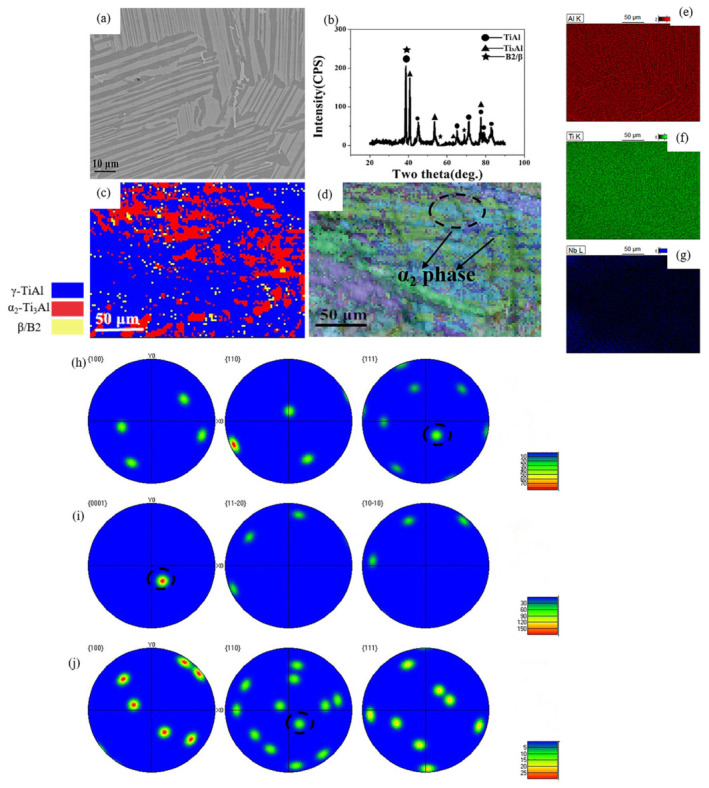
Microstructure, XRD pattern and EBSD characterization of the TiAl-Nb sheet after heat treatment at 1430 °C for 5 min and 1150 °C for 2 h ((**a**) microstructure; (**b**) XRD pattern; (**c**) phase distribution map; (**d**) microstructure under EBSD; (**e**–**g**) distribution of elements in the sheet; (**h**–**j**) pole figure of TiAl phase, Ti_3_Al phase and B2 phase).

Through the analysis of Figure 3a,b, it was found that, after heat treatment at 1430 °C for 5 min and then at 1150 °C for 2 h, combined with Figure 2a, the lamellar structure was refined to a certain extent in the alloy structure. At the γ interface, the α_2_ phase initially nucleated and underwent growth during the heat treatment process. Consequently, after a relatively extended holding period, lamellar α_2_ phases were present at the interface, whereas the α phase that grew within the grains formed a lamellar structure. It should be noted that at higher temperatures, a shorter holding time was required to prevent the growth of lamellar structures. The analysis conducted in conjunction with EBSD (Figure 3c,d) revealed that the lamellar structure within the sheet material remained relatively coarse, with the B2 phase dispersed throughout the microstructure. Moreover, there existed Ti_3_Al phases that had not undergone lamellar transformation in the microstructure, and these plate-like Ti_3_Al phases were primarily situated at the interfaces of the γ phase. Through the analysis of the orientation relationship between the TiAl, Ti_3_Al and B2 phases (Figure 3h–j), the orientation relationship TiAl(111)//Ti_3_Al(0001), TiAl(111)//B2(110) was found.

**Figure 4:**
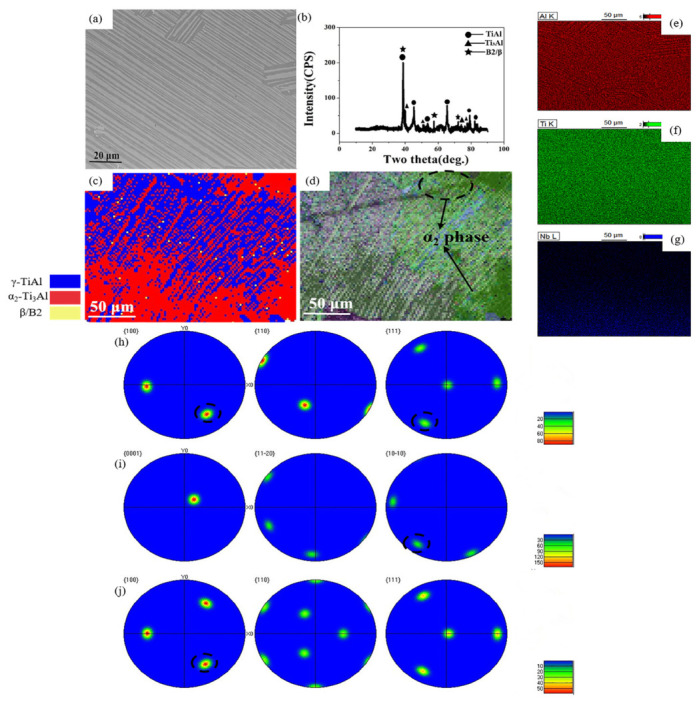
Microstructure, XRD pattern and EBSD characterization of the TiAl-Nb sheet after heat treatment at 1450 °C for 5 min and 1150 °C for 2 h ((**a**) microstructure; (**b**) XRD pattern; (**c**) phase distribution map; (**d**) microstructure under EBSD; (**e**–**g**) distribution of elements in the sheet; (**h**–**j**) pole figure of TiAl phase, Ti_3_Al phase and B2 phase).

Through the analysis of Figure 4a,b, it was determined that, after subjecting the material to heat treatment at 1450 °C for 5 min followed by 1150 °C for 2 h, in comparison with Figure 3 and Figure 4, a relatively coarse lamellar structure was formed in the sheet. Considering the formation mechanism of the lamellar structure, it was observed that the α phase nucleated and grew at the γ/γ interface and the lamellar α_2_ phase was formed at the grain boundary owing to the extended holding time at a high temperature. Simultaneously, the α phase that nucleated within the γ phase also underwent growth, leading to the formation of relatively coarse lamellar structures in the microstructure. Then, it was maintained at a low temperature for a comparatively long period to further uniformly distribute the elements within the structure and refine the lamellar structure of the material to a certain degree. Due to the long holding time at a high temperature, the lamellar structure in the microstructure grew, resulting in relatively coarse lamellar clusters. In conjunction with EBSD analysis, it was discovered that the B2 phase was distributed within the coarse lamellar structure (Figure 4c,d). The combination of Figure 3 further demonstrated that the formation of the α phase at the grain boundaries and within the grains was primarily attributed to the extended heat treatment duration in the β phase region. During this period, the α phase grew, leading to the appearance of relatively coarse lamellar structures in the microstructure, along with the plate-like Ti_3_Al phase at the interfaces. The orientation relationship was as follows (Figure 4h–j): TiAl(111)//Ti_3_Al(10$\bar{1}$0), TiAl(110)//B2(110).

**Figure 5:**
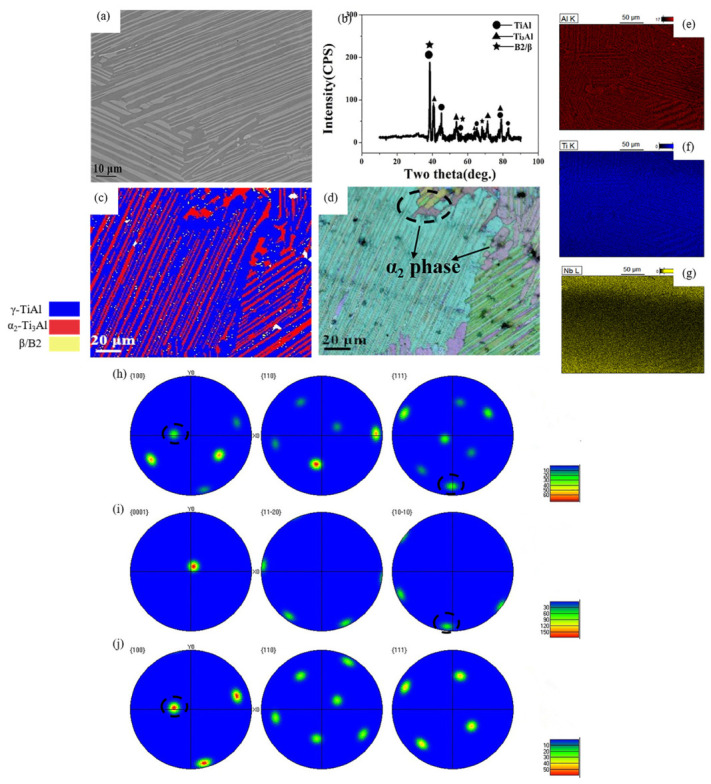
Microstructure, XRD pattern and EBSD characterization of the TiAl-Nb sheet after heat treatment at 1410 °C for 3 min and 1150 °C for 2 h ((**a**) microstructure; (**b**) XRD pattern; (**c**) phase distribution map; (**d**) microstructure under EBSD; (**e**–**g**) distribution of elements in the sheet; (**h**–**j**) pole figure of TiAl phase, Ti_3_Al phase and B2 phase).

Through the analysis of Figure 5a,b, it was found that, after heat treatment at 1410 °C for 3 min and then at 1150 °C for 2 h, the microstructure of the sheet was relatively uniform, and obvious lamellar structures appeared in the sheet microstructure. When compared with the phase composition subsequent to the previous heat treatment, there was no discernible change. The microstructure of the sheet primarily consisted of the TiAl phase, the Ti_3_Al phase, and the B2/β phase. Due to the relatively short high-temperature holding time, less α phase was formed in the alloy structure. Following subsequent heat treatment steps, the α phase did not exhibit growth and instead underwent an ordered transformation, transitioning into the α_2_ phase. Consequently, a relatively fine lamellar structure was formed within the alloy microstructure. Since the α phase formed initially at the γ phase interface and during the holding process, the α phase at the interface grew and transformed into the ordered α_2_ phase, and a lamellar or relatively coarse α_2_ phase appeared at the interface. An analysis of the size of the lamellar clusters in the sheet indicated that smaller lamellar clusters were formed in the microstructure. When combined with EBSD analysis (Figure 5c,d), it was discovered that the phases in the sheet were predominantly the TiAl phase (blue area) and the Ti_3_Al phase (red area), along with a minor quantity of the B2 phase (yellow area). The lamellar structure was relatively fine, with the B2 phase dispersed in the lamellar structure. Meanwhile, the Ti_3_Al phase that had not transformed into the lamellar structure also existed in the structure. It can be found from Figure 5d that the Ti_3_Al phase existed in the form of lamellae at the grain boundaries of the γ phase. This phenomenon was primarily attributed to the fact that, during the heat treatment process, the α_2_ phase nucleated initially at the γ phase grain boundaries and grew as the heat treatment progressed. The phase transformation relationships of TiAl, Ti_3_Al and B2 (Figure 5h–j) were as follows: TiAl(111)//Ti_3_Al(10$\bar{1}$0), TiAl(100)//B2(100).

**Figure 6:**
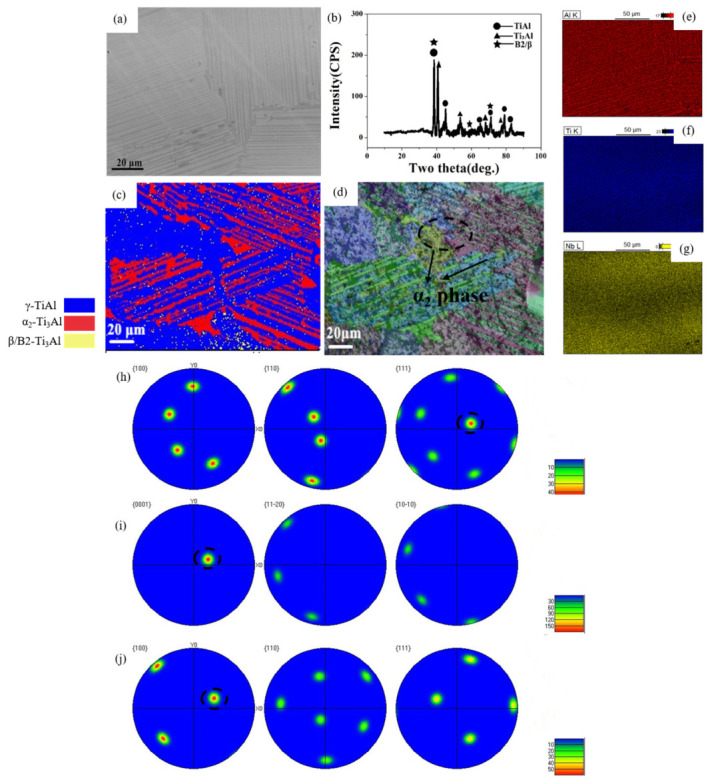
Microstructure, XRD pattern and EBSD characterization of the TiAl-Nb sheet after heat treatment at 1430 °C for 3 min and 1150 °C for 2 h ((**a**) microstructure; (**b**) XRD pattern; (**c**) phase distribution map; (**d**) microstructure under EBSD; (**e**–**g**) distribution of elements in the sheet; (**h**–**j**) pole figure of TiAl phase, Ti_3_Al phase and B2 phase).

After the two-step heat treatment at 1430 °C for 3 min and at 1150 °C for 2 h (Figure 6a,b), TiAl phase, Ti_3_Al phase and B2/β phase were the main constituent phases of the alloy, and the lamellar structure was further refined, combined with Figure 3. Owing to the relatively brief holding duration during high-temperature heat treatment, only a limited quantity of the α phase nucleated and grew at the interface of the γ phase. The α phase predominantly nucleated within the γ phase and, following an ordered transformation, developed a relatively fine lamellar structure. There was no obvious lamellar Ti_3_Al phase in the sheet. The microstructure was mainly composed of a γ/α_2_ lamellar structure and γ phase. In conjunction with the EBSD analysis (Figure 6c,d), it was discovered that the lamellar structure within the sheet metal was relatively refined, and the B2 phase was distributed within the lamellar structure. The α_2_ phase exhibited less nucleation at the γ phase interface. During the heat treatment process, the α phase predominantly nucleated within the γ phase and ultimately formed a relatively fine lamellar structure. TiAl, Ti_3_Al and B2 phases had the following orientation relationships (Figure 6h–j): TiAl(111)//Ti_3_Al(0001), TiAl(111)//B2(100).

**Figure 7:**
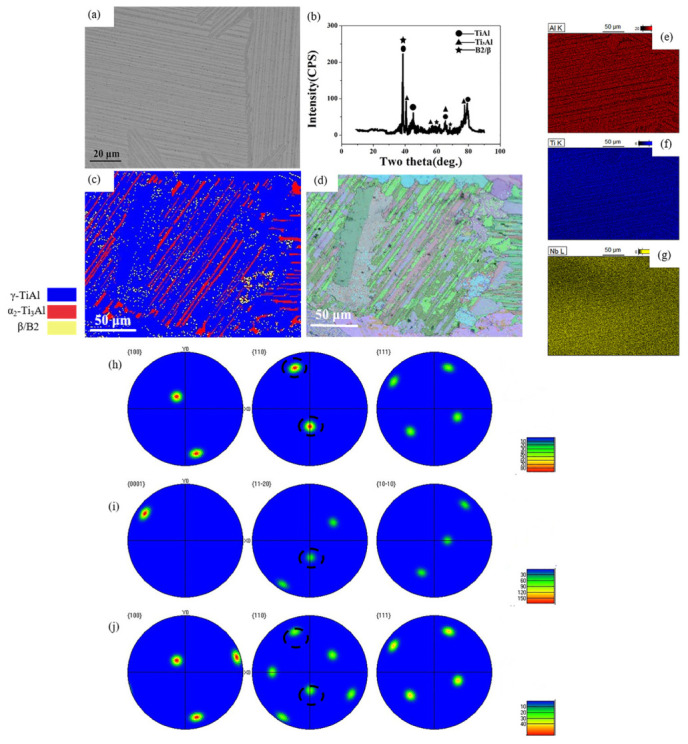
Microstructure, XRD pattern and EBSD characterization of the TiAl-Nb sheet after heat treatment at 1450 °C for 3 min and 1150 °C for 2 h ((**a**) microstructure; (**b**) XRD pattern; (**c**) phase distribution map; (**d**) microstructure under EBSD; (**e**–**g**) distribution of elements in the sheet; (**h**–**j**) pole figure of TiAl phase, Ti_3_Al phase and B2 phase).

Through the analysis of Figure 7a,b, it was observed that when the material was held at 1450 °C for 3 min and then at 1150 °C for 2 h, the lamellar structure in the microstructure of the sheet was relatively finer compared with that in Figure 4. By analyzing the formation mechanism of the lamellar structure, it could be observed that, owing to the relatively short high-temperature holding time, the α phase nucleated within the γ phase and at the γ/γ interface. Due to the brief retention time within the β phase region, the α phase failed to grow, followed by an ordered transformation, which led to the formation of a relatively fine lamellar structure. In combination with EBSD analysis, it was found that the lamellar structure in the sheet metal was relatively fine and was composed of the B2 phase that was dispersedly distributed. Owing to the brief holding time within the β phase region, despite the formation of α phase nuclei at the grain boundaries and inside the grains, the formed α phase failed to grow. Consequently, subsequent to the ordering transformation, a fine lamellar structure emerged in the microstructure (Figure 7c,d). The orientation relationship was as follows (Figure 7h–j)): TiAl(110)//Ti_3_Al(11$\bar{2}$0), TiAl(110)//B2(110).

The results demonstrated that under short β-phase holding conditions (1410 °C × 3 min, 1430 °C × 3 min, and 1450 °C × 3 min), the average lamellar spacing values were approximately 98 nm, 105 nm, and 115 nm. These values were significantly smaller than those obtained for the corresponding 5 min holding conditions (210 nm, 195 nm, 230 nm). The EBSD grain boundary analysis results were consistent with these values, confirming that reducing the high-temperature holding time effectively suppressed α-phase growth and refined the lamellar spacing by more than 50%. The average EBSD confidence index ranged from 0.32 to 0.41 across all samples. The γ phase fraction varied between 64% and 72%, the α_2_ phase fraction ranged from 23% to 30%, and the B2 phase fraction remained between 5% and 6%. The short-time holding groups (3 min) exhibited a slightly higher γ-phase fraction (72%), corresponding to the finer lamellar structure.

Based on the aforementioned data, it was discovered that, when the heat treatment processes were 1410 °C for 3 min + 1150 °C for 2 h, 1430 °C for 3 min + 1150 °C for 2 h, and 1430 °C for 3 min + 1150 °C for 2 h, the microstructure of the TiAl—Nb alloy sheet consisted of the γ phase and a relatively fine γ/α_2_ lamellar structure.

### 3.2. TEM and HRTEM Analysis After Heat Treatment at Different Temperatures

Through TEM and HRTEM analyses of the microstructures after heat treatment at various temperatures, the orientation relationships between each phase within the microstructures were determined. The microstructures exhibiting a preferred orientation were selected, which, in turn, provided a theoretical foundation for the selection of the heat treatment process.

**Figure 8:**
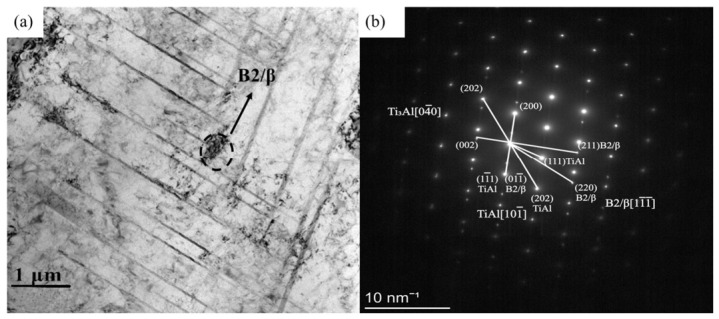
TEM image and diffraction pattern of the TiAl-Nb sheet structure after heat treatment at 1410 °C for 3 min and 1150 °C for 2 h ((**a**) microstructure; (**b**) diffraction pattern).

After the TiAl-Nb sheet underwent heat treatment at 1410 °C for 3 min and subsequently at 1150 °C for 2 h, the microstructure of the sheet predominantly consisted of the matrix γ-TiAl, lamellar α_2_-Ti_3_Al, and B2/β phases. The α_2_ phase was dispersed within the γ phase in the form of fine needle-like structures, and the B2/β phase was primarily distributed within the microstructure in the form of ellipsoids, as depicted in Figure 8a. As shown in Figure 8b, the mismatch between the TiAl and Ti_3_Al phases was 0.08, and they were semi-coherent interfaces. Therefore, Ti_3_Al and the matrix TiAl phase were combined in the form of a semi-coherent interface. The mismatch between B2/β and TiAl phases was 0.008, with the two combined in the form of a coherent interface.

**Figure 9:**
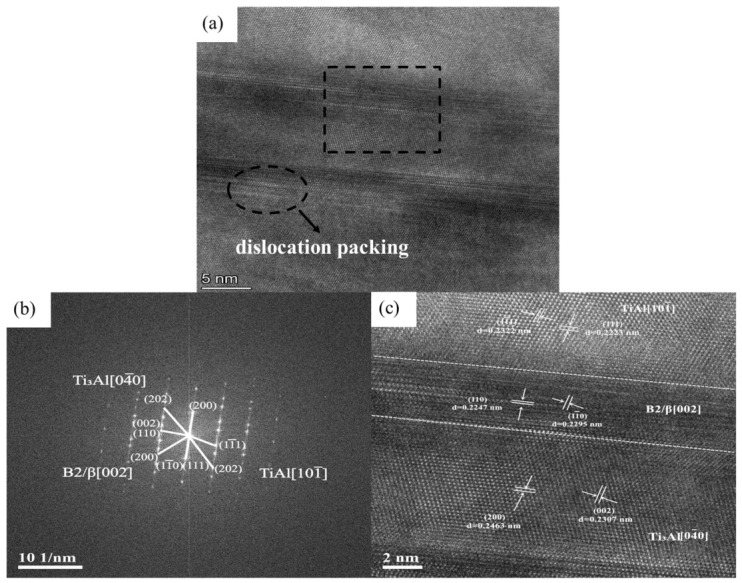
HRTEM images of Ti_3_Al, B2/β and TiAl interfaces after heat treatment at 1410 °C for 3 min and 1150 °C for 2 h ((**a**) HRTEM image; (**b**) selected area Fourier transform image; (**c**) selected area inverse Fourier transform image).

Figure 9a–c shows the HRTEM image of the TiAl-Nb sheet after heat treatment at 1410 °C for 3 min and 1150 °C for 2 h. After analysis, it was determined that the following crystallographic relationships existed among the interfaces of the γ-TiAl, α_2_-Ti_3_Al, and B2/β phases:

TiAl[10$\bar{1}$]//Ti_3_Al[0$\bar{4}$0]//B2/β[002]

(1$\bar{1}$1)TiAl//(002)Ti_3_Al//(1$\bar{1}$0)B2/β

(111)TiAl//(200)Ti_3_Al//(110)B2/β

The mismatch degree between (1$\bar{1}$1)TiAl and (002)Ti_3_Al was calculated to be 0.15%. TiAl and Ti_3_Al were well combined in the form of a coherent interface, and the two phases were well combined without a fixed or preferred crystallographic orientation. By calculating the mismatch degree of the (111)TiAl and the (200)B2/β, it was found that the mismatch degree between the two was 0.27%. The two also existed in the form of a coherent interface, and there was no fixed or preferred crystallographic orientation between them. Upon analysis of Figure 9c, it was evident that there was distinct dislocation packing between the Ti_3_Al phase, the B2/β phase, and the TiAl phase. This indicated that the presence of the Ti_3_Al phase and the B2/β phase impeded the movement of dislocations, leading to dislocation packing and exerting a certain strengthening effect on the sheet. Simultaneously, the TiAl phase, Ti_3_Al phase, and B2/β phase coexisted in the form of coherent interfaces, and the degree of mismatch was extremely low, suggesting that the interfacial surface energy was low. Consequently, it was more feasible to obtain a sheet structure containing a relatively large quantity of the γ(TiAl) phase.

**Figure 10:**
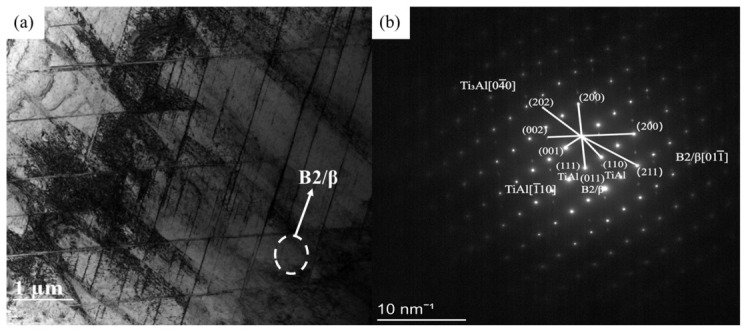
TEM image and diffraction pattern of the TiAl-Nb sheet structure after heat treatment at 1430 °C for 3 min and 1150 °C for 2 h ((**a**) microstructure; (**b**) diffraction pattern).

After the TiAl-Nb sheet was maintained at 1430 °C for 3 min and subsequently at 1150 °C for 2 h, the microstructure of the sheet primarily comprised the matrix γ-TiAl, lamellar α_2_-Ti_3_Al, and ellipsoidal B2/β phases. The microstructure was predominantly lamellar, as depicted in Figure 10a. The B2/β phases were dispersed within the relatively fine lamellar microstructure. As indicated in Figure 10b, the mismatch between the TiAl and Ti_3_Al phases was 0.08. The two were semi-coherent interfaces and they combined well. The mismatch between the B2/β and TiAl phases was 0.008, and the two combined in the form of a coherent interface.

**Figure 11:**
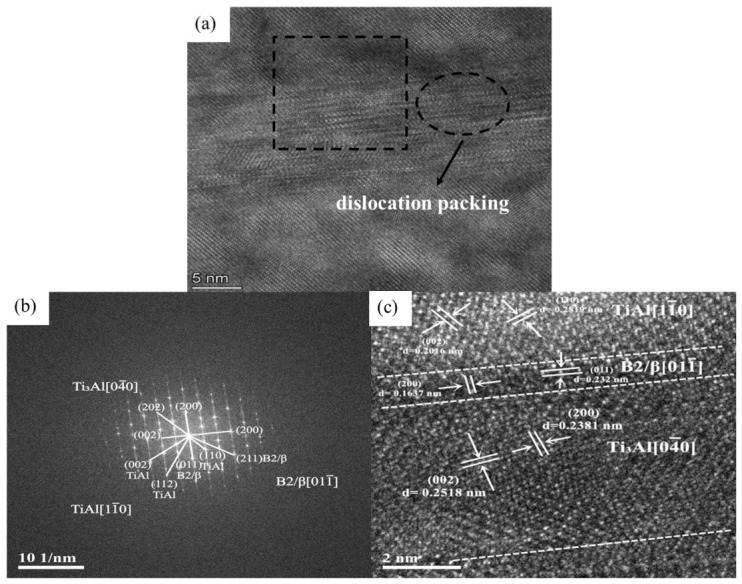
HRTEM images of Ti_3_Al, B2/β and TiAl interfaces after heat treatment at 1430 °C for 3 min and 1150 °C for 2 h ((**a**) HRTEM image; (**b**) selected area Fourier transform image; (**c**) selected area inverse Fourier transform image).

Figure 11a presents the HRTEM image of the TiAl-Nb sheet subsequent to being held at 1430 °C for 3 min and then at 1150 °C for 2 h. The following crystallographic relationships were present among the interfaces of the γ-TiAl, α_2_-Ti_3_Al, and B2/β phases:

TiAl[1$\bar{1}$0]//Ti_3_Al[0$\bar{4}$0]//B2/β[01$\bar{1}$]

(002)TiAl//(200)Ti_3_Al//(200)B2/β

(110)TiAl//(002)Ti_3_Al//(011)B2/β

The degree of mismatch between (002)TiAl and (200)Ti_3_Al was calculated to be 0.18. According to classical interface crystallography, a misfit δ < 0.05 is defined as a coherent interface, 0.05 ≤ δ < 0.25 as a semi-coherent interface (with misfit dislocations), and δ ≥ 0.25 as an incoherent interface. TiAl and Ti_3_Al were tightly combined in the form of a semi-coherent interface, and there was no fixed or preferred crystallographic orientation between the two phases. Through the calculation of the mismatch degree between the (002)TiAl and the (200)B2/β, it was found that the mismatch between the two planes was 0.187. They also existed in the form of a semi-coherent interface, and there was no fixed or preferred crystallographic orientation between them. In Figure 11a, it could be observed that there was dislocation packing at the interfaces between the Ti_3_Al phase and the B2/β phase, as well as between the Ti_3_Al phase and the TiAl phase. Both the Ti_3_Al phase and the B2/β phase impeded the movement of dislocations. This impedance resulted in dislocation packing at the interfaces, consequently exerting a certain strengthening effect on the sheet. However, when compared with the microstructure obtained after holding at 1410 °C for 3 min and subsequently at 1150 °C for 2 h, the phases predominantly existed in the form of semi-coherent interfaces, and the mismatch was relatively significant. This made it challenging to acquire a microstructure with a higher content of the γ(TiAl) phase after heat treatment.

**Figure 12:**
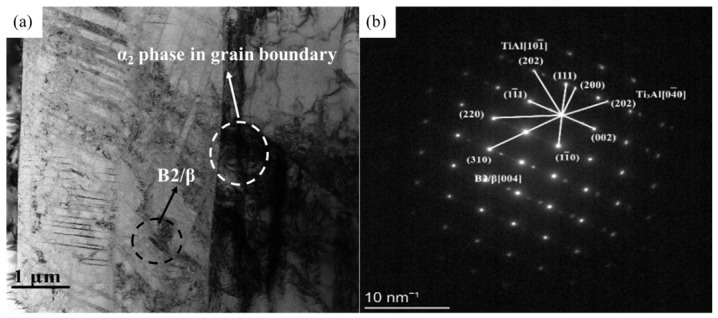
TEM image and diffraction pattern of the TiAl-Nb sheet structure after heat treatment at 1450 °C for 3 min and 1150 °C for 2 h ((**a**) microstructure; (**b**) diffraction pattern).

After the TiAl-Nb sheet underwent heat treatment at 1450 °C for 3 min and subsequently at 1150 °C for 2 h, as observed from Figure 12a, the α_2_ phase within the grains was relatively fine. Compared with the α_2_ phase within the grains, the α_2_ phase existing at the grain boundaries was coarser. The primary cause of this phenomenon was that, during the heat treatment process, the α phase initially nucleated and grew at the grain boundaries, followed by an ordered transformation, and it persisted at the grain boundaries. The mismatch between the TiAl and Ti_3_Al phases was 0.0004, and the two were in the form of a coherent interface. The mismatch between the B2/β and TiAl phases was 0.01, and the two combined in the form of a coherent interface.

**Figure 13:**
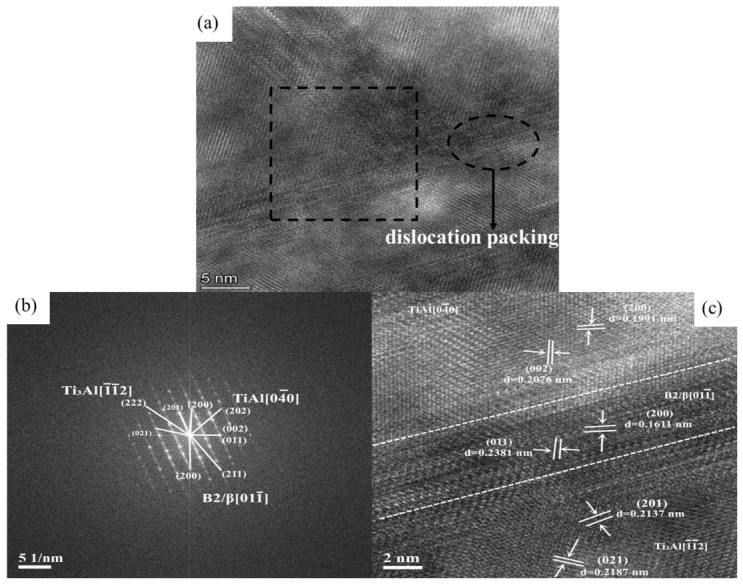
HRTEM images of Ti_3_Al, B2/β and TiAl interfaces after heat treatment at 1450 °C for 3 min and 1150 °C for 2 h ((**a**) HRTEM image; (**b**) selected area Fourier transform image; (**c**) selected area inverse Fourier transform image).

Through the analysis of Figure 13, it was found that the following crystallographic relationships existed among the γ-TiAl, α_2_-Ti_3_Al and B2/β phase interfaces:

TiAl [0$\bar{4}$0]//Ti_3_Al[$\bar{1}\bar{1}$2]//B2/β[01$\bar{1}$]

(200)TiAl//(201)Ti_3_Al//(200)B2/β

(002)TiAl//(021)Ti_3_Al//(011)B2/β

The mismatch between the (002)TiAl and (201)Ti_3_Al phases was 5%. A semi-coherent interface was formed between TiAl and Ti_3_Al, and the two phases combined well without a fixed or preferred crystallographic orientation. The mismatch between (002)TiAl and (200)B2/β was 22.4%, and they also existed in the form of a semi-coherent interface without a fixed or preferred crystallographic orientation. In Figure 13a, it was found that there was also obvious dislocation packing between the Ti_3_Al phase and the B2/β phase as well as the TiAl phase, indicating that the presence of the Ti_3_Al phase and the B2/β phase hindered dislocation movement and played a certain strengthening role in the sheet. However, when compared with the microstructure obtained after holding at 1410 °C for 3 min and subsequently at 1150 °C for 2 h, the interfaces between the phases predominantly existed in the form of semi-coherent interfaces. Among these interfaces, the mismatch between the B2/β phase and the matrix TiAl phase was relatively high. As a result, it was difficult for the sheet to acquire a microstructure containing a substantial amount of the γ(TiAl) phase after heat treatment.

### 3.3. Analysis of the Orientation Relationship Between TiAl and Ti_3_Al After Heat Treatment at Different Temperatures

To further provide a basis for the selection of heat treatment processes, this paper employs the transformation matrix method to investigate the crystallographic orientation relationship between the TiAl phase and the Ti_3_Al phase in the microstructure of TiAl-Nb sheets after different heat treatment processes and consequently determine the preferred orientation relationship of the two phases in the TiAl-Nb alloy sheets. The principle of the matrix method involves calculating the transformation matrices B and A for each crystal orientation relationship. If the absolute values of the nine elements in transformation matrices A and B are identical, despite differences in their order and position, it can be concluded that these two crystallographic orientation relationships belong to the same type.

According to the research [22], the crystal plane (hkl) could be expressed as G^*^_hkl_ = ha_1_^*^ + ka_2_^*^ + la_3_^*^, and the vector of the normal direction of the (hkl) crystal plane is I_uvw_ = ua_1_ + va_2_ + wa_3_. The relationship between G^*^_hkl_ = ha_1_^*^ + ka_2_^*^ + la_3_^*^ and I_uvw_ = ua_1_ + va_2_ + wa_3_ is as follows:

(1) $$\left[\begin{array}{c} h\\ k\\ l \end{array}\right]=G\left[\begin{array}{c} u\\ v\\ w \end{array}\right]$$

(2) $$\left[\begin{array}{c} u\\ v\\ w \end{array}\right]=G^{-1}\left[\begin{array}{c} h\\ k\\ l \end{array}\right]$$

(3) $$G=\left(\begin{array}{ccc} a^{2} & \mathrm{abcos}\gamma & \mathrm{accos}\beta \\ \mathrm{abcos}\gamma & b^{2} & \mathrm{bccos}\alpha \\ \mathrm{accos}\beta & \mathrm{bccos}\alpha & c^{2} \end{array}\right)$$

This formula is applicable to all crystal systems. G represents the transformation matrix when the direct-space basis vectors a_1_, a_2_, a_3_ of the crystal are expressed in relation to the reciprocal-space basis vectors a_1_^*^, a_2_^*^, a_3_^*^; G^−1^ denoted the inverse matrix of the transformation matrix G; a, b, and c are the lattice constants, while α, β, and γ are the inter-axial angles of the crystal [23].

The lattice constant of Ti_3_Al is: a = b = 5.77 Å, c = 4.62 nm Å, α = β = 90°, γ = 120°. The lattice constant of TiAl is: a = b = 4.001 Å, c = 4.071 Å, α = β = γ = 90°. The above parameters are substituted into (3).

Based on the parallel relationship of crystal directions [u_2_′v_2_′w_2_′]//[u_2_v_2_w_2_] and Equation (1), the parallel relationship of the second set of crystal planes (h_2_′k_2_′l_2_′)//(h_2_k_2_l_2_) could be derived. Similarly, based on the parallel relationship of crystal planes (h_1_′k_1_′l_1_′)//(h_1_k_1_l_1_) and Equation (2), the parallel relationship of crystal directions [u_1_′v_1_′w_1_′]//[u_1_v_1_w_1_] could be derived. Consequently, the following three sets of parallel relationships can be obtained:

$$\{\begin{array}{c} \left(h_{1}\prime k_{1}\prime l_{1}\prime )//(h_{1}k_{1}l_{1}\right)\\ \left(h_{2}\prime k_{2}\prime l_{2}\prime )//(h_{2}k_{2}l_{2}\right)\\ \left(h_{3}\prime k_{3}\prime l_{3}\prime )//(h_{3}k_{3}l_{3}\right) \end{array}$$

The orientation relationships between the Ti_3_Al and TiAl phases under different heat treatment conditions are summarized in Table 2.

The aforementioned bit-related relationship converts into the following matrix relationship:

(4) $$\left[\begin{array}{c} u\prime \\ v\prime \\ w\prime \end{array}\right]=B\left[\begin{array}{c} u\\ v\\ w \end{array}\right]\;\mathrm{and}\;\left[\begin{array}{c} h\prime \\ k\prime \\ l\prime \end{array}\right]=A\left[\begin{array}{c} h\\ k\\ l \end{array}\right]$$

A and B are transposed inverse matrices of each other, and B is the transformation matrix:

(5) $$B=\left[\begin{array}{ccc} h_{1}\prime & k_{1}\prime & l_{1}\prime \\ h_{2}\prime & k_{2}\prime & l_{2}\prime \\ h_{3}\prime & k_{3}\prime & l_{3}\prime \end{array}\right]\left[\begin{array}{ccc} \frac{d_{1}}{d_{1}\prime } & 0 & 0\\ 0 & \frac{d_{2}}{d_{2}\prime } & 0\\ 0 & 0 & \frac{d_{3}}{d_{3}\prime } \end{array}\right]\left[\begin{array}{ccc} h_{1} & k_{1} & l_{1}\\ h_{2} & k_{2} & l_{2}\\ h_{3} & k_{3} & l_{3} \end{array}\right]$$

d_1_, d_2_, d_3_ are respectively the interplanar spacings of the (h_1_k_1_l_1_), (h_2_k_2_l_2_) and (h_3_k_3_l_3_) crystal planes of the TiAl phase, while d_1_′, d_2_′, d_3_′ are respectively the interplanar spacings of the (h_1_′k_1_′l_1_′), (h_2_′k_2_′l_2_′) and (h_3_′k_3_′l_3_′) crystal planes of the Ti_3_Al phase.

As shown in Table 3, the calculation results indicate that, after heat treatment at 1430 °C and 1450 °C, there was no fixed or preferred crystal orientation relationship between the Ti_3_Al phase and the TiAl matrix phase. After being maintained at 1410 °C for 3 min and subsequently at 1150 °C for 2 h, the Ti_3_Al phase and the TiAl matrix phase exhibited a preferred orientation relationship, specifically Ti_3_Al[0$\bar{4}$0]//TiAl[10$\bar{1}$], (002)Ti_3_Al//(1$\bar{1}$1)TiAl.

The variations in the Ti_3_Al/TiAl orientation relationships measured under different heat-treatment conditions reflected differences in variant selection during the α→α_2_ transformation. Short β-phase holding conditions (1410 °C × 3 min) promoted preferential α-phase nucleation at γ grain boundaries, resulting in the strong selection of specific orientation variants such as (002)Ti_3_Al//(111)TiAl, with a frequency of 72%. In contrast, higher temperatures or longer holding times promoted intragranular homogeneous nucleation, leading to a more random distribution of orientation variants.

Based on previous research findings regarding the mismatch, the interface of (002)Ti_3_Al//(1$\bar{1}$1)TiAl not only exhibited a preferred orientation but also had a relatively small degree of mismatch. Owing to heat treatment within the β phase region, during the heat-treatment process, the α phase nucleated and grew at the interface between the γ phase and the β phase and, subsequently, underwent an ordered transformation to form the α_2_ phase. When the mismatch between the two phases was lower and they exhibited a favorable crystallographic matching degree, a greater quantity of γ(TiAl) and α_2_/γ lamellar structures could be formed within the matrix, and the formed lamellar structures were relatively fine.

In summary, by combining EBSD-based variant frequency statistics with matrix method analysis, we elucidated the variant selection rules during the B2/β→α→α_2_ + γ transformation under different heat-treatment paths. It was shown that a short β-phase holding favored the formation of low interfacial energy-preferred orientations, whereas higher temperatures or longer holding times lead to variant randomization.

## 4. Conclusions

Based on the microstructure analysis of the Ti-47Al-5Nb (at%) alloy sheet subsequent to heat treatment, the following conclusions were derived:

(1) As the heat treatment temperature increased, the microstructure of the alloy sheet primarily consisted of a γ/α_2_ lamellar structure. When the heat treatment was conducted at a relatively high temperature for a brief period, a greater number of α_2_/γ lamellar structures formed within the microstructure. Nevertheless, as the holding time prolonged, coarser lamellar structures developed in the microstructure.
(2) After heat treatment at 1410 °C for 3 min and then at 1150 °C for 2 h, at 1430 °C for 3 min and then at 1150 °C for 2 h, and at 1450 °C for 3 min and then at 1150 °C for 2 h, the microstructure of the TiAl-Nb alloy sheet was composed of relatively fine lamellar structures.
(3) After being maintained at 1410 °C for 3 min and then at 1150 °C for 2 h, the TiAl phase, Ti_3_Al phase, and B2/β phase coexisted in the form of coherent interfaces with an extremely low mismatch, and the interfacial energy between the phases was small. Consequently, it was more feasible to obtain an alloy microstructure with a higher content of the γ(TiAl) phase.
(4) After conducting an analysis using the matrix method, it was discovered that the TiAl phase and the Ti_3_Al phase, which were held at 1410 °C for 3 min and subsequently at 1150 °C for 2 h, exhibited obvious preferred orientations, namely Ti_3_Al[0$\bar{4}$0]//TiAl [10$\bar{1}$], (002)Ti_3_Al//(1$\bar{1}$1)TiAl.
(5) These quantitative data demonstrated that a short β-phase holding (3 min) followed by a long α + γ two-phase holding was an effective process route for obtaining a fine lamellar structure.

## 5. Future Work

Although the present study indirectly demonstrates the potential improvement in mechanical properties through quantitative microstructural characterization (lamellar spacing, phase fraction, interfacial misfit, etc.), direct mechanical validation is still missing. In the next step, we will systematically measure the room-temperature and high-temperature tensile strength, fracture toughness, creep, and fatigue properties of the alloy under different heat treatment conditions and establish a quantitative relationship model by integrating the orientation relationship and interface structure findings reported here.

## Figures and Tables

**Table 1:** Parameters of heat treatment microstructure control process.

Number	Heat Treatment Microstructure Control
1	1410 °C × 5 min + 1150 °C × 2 h
2	1430 °C × 5 min + 1150 °C × 2 h
3	1450 °C × 5 min + 1150 °C × 2 h
4	1410 °C × 3 min + 1150 °C × 2 h
5	1430 °C × 3 min + 1150 °C × 2 h
6	1450 °C × 3 min + 1150 °C × 2 h

**Table 2:** The orientation relationships of Ti_3_Al/TiAl interface after heat treatment at different temperatures.

Heat Treatment Process	[u’v’w’]Ti_3_Al//[uvw]TiAl	(h’k’l’)Ti_3_Al//(hkl)TiAl
1410 °C 3 min + 1150 °C 2 h	Ti_3_Al[0$\bar{4}$0]//TiAl[10$\bar{1}$]	(002)Ti_3_Al//(1$\bar{1}$1)TiAl
1430 °C 3 min + 1150 °C 2 h	Ti_3_Al[0$\bar{4}$0]//TiAl[1$\bar{1}$0]	(200)Ti_3_Al//(002)TiAl
1450 °C 3 min + 1150 °C 2 h	Ti_3_Al[$\bar{1}\bar{1}$2]//TiAl[0$\bar{4}$0]	(201)Ti_3_Al//(200)TiAl

**Table 3:** The parallel planes and transformation matrix between Ti_3_Al and TiAl.

Heat Treatment Process	(h’k’l’)Ti3Al//(hkl)TiAl	Transformation Matrix B	Transformation Matrix A
1410 °C 3 min + 1150 °C 2 h	(002)Ti3Al//(1$\bar{1}$1)TiAl; ($\bar{1}\bar{2}$0)Ti3Al//(10$\bar{1}$)TiAl; ($\bar{8}$00)//(1$\bar{2}\bar{1}$)TiAl	$\left[\begin{array}{ccc} 5.24 & -10.49 & -5.24\\ -4.02 & 0.99 & 2.03\\ -7.97 & 7.97 & -7.97 \end{array}\right]$	$\left[\begin{array}{ccc} -0.05 & -0.10 & -0.05\\ -0.25 & -0.16 & 0.08\\ -0.03 & 0.03 & -0.03 \end{array}\right]$
1430 °C 3 min + 1150 °C 2 h	(200)Ti3Al//(002)TiAl; (1$\bar{2}$0)Ti3Al//(1$\bar{1}$0)TiAl; (008)Ti3Al//($\bar{2}$00)TiAl	$\left[\begin{array}{ccc} 0 & 0 & 3.26\\ -0.30 & 0.30 & 1.63\\ -55.13 & 0 & 0 \end{array}\right]$	$\left[\begin{array}{ccc} 0 & -1.68 & 0.31\\ 0 & 3.36 & 0\\ -0.02 & -0.02 & 0 \end{array}\right]$
1450 °C 3 min + 1150 °C 2 h	(201)Ti3Al//(200)TiAl; (225)Ti3Al//(0$\bar{4}$0)TiAl; ($\bar{3}$51)Ti3Al//(008)TiAl	$\left[\begin{array}{ccc} 5.61 & 0 & 3.66\\ 13.61 & -7.76 & 18.29\\ -3.42 & -19.41 & 3.65 \end{array}\right]$	$\left[\begin{array}{ccc} 0.30 & 0.88 & -0.19\\ -0.06 & -0.03 & 0.08\\ -0.02 & 0.04 & 0.03 \end{array}\right]$

## Data Availability

The original contributions presented in this study are included in the article. Further inquiries can be directed to the corresponding authors.
